# Hypothesis: The Vestibular and Cerebellar Basis of the Mal de Debarquement Syndrome

**DOI:** 10.3389/fneur.2018.00028

**Published:** 2018-02-05

**Authors:** Bernard Cohen, Sergei B. Yakushin, Catherine Cho

**Affiliations:** ^1^Department of Neurology, Icahn School of Medicine at Mount Sinai, New York, NY, United States; ^2^Department of Neurology, NYU School of Medicine, New York, NY, United States; ^3^Department of Otolaryngology, NYU School of Medicine, New York, NY, United States

**Keywords:** vestibular-only neurons, nodulus, baclofen, rocking, swaying, bobbing, gravity, orientation vector

## Abstract

The Mal de Debarquement syndrome (MdDS) generally follows sea voyages, but it can occur after turbulent flights or spontaneously. The primary features are objective or perceived continuous rocking, swaying, and/or bobbing at 0.2 Hz after sea voyages or 0.3 Hz after flights. The oscillations can continue for months or years and are immensely disturbing. Associated symptoms appear to be secondary to the incessant sensation of movement. We previously suggested that the illness can be attributed to maladaptation of the velocity storage integrator in the vestibular system, but the actual neural mechanisms driving the MdDS are unknown. Here, based on experiments in subhuman primates, we propose a series of postulates through which the MdDS is generated: (1) The MdDS is produced in the velocity storage integrator by activation of vestibular-only (VO) neurons on either side of the brainstem that are oscillating back and forth at 0.2 or 0.3 Hz. (2) The groups of VO neurons are driven by signals that originate in Purkinje cells in the cerebellar nodulus. (3) Prolonged exposure to roll, either on the sea or in the air, conditions the roll-related neurons in the nodulus. (4) The prolonged exposure causes a shift of the pitch orientation vector from its original position aligned with gravity to a position tilted in roll. (5) Successful treatment involves exposure to a full-field optokinetic stimulus rotating around the spatial vertical countering the direction of the vestibular imbalance. This is done while rolling the head at the frequency of the perceived rocking, swaying, or bobbing. We also note experiments that could be used to verify these postulates, as well as considering potential flaws in the logic. Important unanswered questions: (1) Why does the MdDS predominantly affect women? (2) What aspect of roll causes the prolongation of the tilted orientation vector, and why is it so prolonged in some individuals? (3) What produces the increase in symptoms of some patients when returning home after treatment, and how can this be avoided? We also posit that the same mechanisms underlie the less troublesome and shorter duration Mal de Debarquement.

## Definitions

**Table:** 

Brain fog	disruption of ability to think clearly
Classic MdDS	MdDS arising from travel on the sea or in the air
Spontaneous MdDS	MdDS generally arising after exposure to motion, but without known exposure to sea or air travel
Dutch roll	flutter of wings and fuselage of aircraft when banking in turbulent weather
Gravity pulling	Sensation of being pulled in one particular direction MdDS
Pitch orientation vector	Vector generally directed toward the spatial vertical that underlies balance
Rocking	movement or sensation of movement forward and back, generally at 0.2 Hz
Swaying	movement or sensation of movement from side-to-side, frequently with a rotary component
Bobbing	sensation of vertical movement of the head and body, generally not associated with actual movement
Roll while rotating	rotation of monkeys in darkness about a vertical axis at 60°/s for several hours while oscillating ±20° at 0.1 Hz in roll
Pitch while rotating	rotation of monkeys in darkness at 60°/s for several hours around a vertical axis while oscillating at 0.1 Hz at ±20° in pitch at 0.1 Hz

## Introduction

The Mal de Debarquement Syndrome (MdDS) is composed of primary and secondary symptoms. The major primary effects are the continuous rocking, swaying, bobbing, or continuous sensations of these phenomena at 0.2 Hz after being on the sea or 0.3 Hz after turbulent flight (1, 2). These symptoms cease briefly when riding in a car (1–8). The patients also frequently experience a sensation of “pulling” in specific directions (“gravity pulling”) (2). The MdDS pathology can be extended over months or years, giving a sense of continuous oscillatory motion that seriously affects the lives of the sufferers, who are predominantly middle-aged women. The incessant rocking, swaying, and/or bobbing are frequently associated with a host of symptoms such as brain fog, sensitivity to sound and fluorescent lights, headaches, inability to work, depression, and suicidal tendencies (2, 4, 6, 9, 10).

Neither the cause for nor the changes in neural activity producing the MdDS are known (4, 6, 7), but there have been many hypotheses to explain the source of the MdDS. These include “vestibular adaptation” or “defective readaptation” (3–5, 11), although the specifics of the vestibular adaptation were not detailed. The MdDS has also been attributed to various cerebral processes that involve the vestibular projections to the cerebral cortex (7), overactivity of the hippocampus and entorhinal cortex (7), interaction of cerebral processes (7, 12), increased sensitivity of the cerebrospinal pathways (13), and modulation of general activity following loss of gray matter in the prefrontal cortex, entorhinal cortex, and cerebellum (14). These studies led to the use of transcranial magnetic stimulation that produced transient relief of symptoms (7, 13) but not to prolonged disappearance of the symptoms of the MdDS. It has also been proposed that the MdDS is a vestibular analog of the Charles Bonnet syndrome, with the recurrent oscillations reflecting a loss of vestibular input (15). However, the function of the semicircular canals is generally intact in these individuals. Thus, there is an inherent difference between the Charles Bonnet sufferers, in whom the loss of vision is the precipitating cause for the visual hallucinations (16, 17) and the MdDS where there is no loss of vestibular function.

There have also been attempts to quell the symptoms with medication. These medications include GABA_a_ agonists like diazepam or clonazepam, nortriptyline, verapamil, and topiramate. Generally, these produced only mild or moderate reduction of symptoms (4, 12). Vestibular rehabilitation has generally been unsuccessful in stopping the sensed or actual movements of the MdDS, and extensive medical workups that include MRI’s, tests of vestibular function with video nystagmography, and tests of auditory and otolith function have all been normal. It has been estimated that the costs of these normal tests extend into the thousands of dollars. Until recently, there has been no successful treatment of the MdDS, nor is it clear how and where the process is generated in the central nervous system.

In 2014, Dai et al. (1) introduced the first successful treatment of the MdDS, and several hundred patients have been successfully treated since that time (2). However, the succession of the neural events that produce the MdDS is still relatively obscure. In this paper, we present a hypothesis composed of a number of postulates that presumably will explain the neural basis of the internal structure responsible for this condition. Other than the proposed maladaptation of the velocity storage integrator in the vestibulo-ocular reflex (VOR) (1), there is no theory detailing the neural pathways involved in generating the incessant rocking, swaying, and/or bobbing or a sense of these oscillations that are the main features of the illness (1). Though important questions remain, it is the first such analysis of the vestibular and cerebellar components that we believe are responsible for generating the MdDS.

The vestibular basis for the treatment came from experiments on the monkey (18). The monkeys were rotated for several hours in darkness while oscillating in roll. Afterward, the animals had horizontal spontaneous nystagmus and unusual vertical positional nystagmus when their heads were rolled to either side. The quick phases of the vertical nystagmus were upward when the head was rolled to one side and downward when the head was rolled to the other side (18), similar to the vertical positional nystagmus with head roll in the MdDS patients (1). These changes persisted for about 18 h and were never induced in monkeys that had very short VOR time constants, i.e., in animals that had appropriate vestibular responses to angular acceleration but no velocity storage. This was modeled, and it was concluded that the positional nystagmus had been produced by cross-coupling of the pitch orientation eigenvector that had been shifted in roll after exposure to roll while rotating. A similar shift in the pitch orientation vector was not produced by pitch while rotating. It was presumed that the pitch while rotating only strengthened the pitch orientation vector in its alignment along the spatial vertical (see Ref. (19–22) for a more complete description of the characteristics of velocity storage). The striking similarities between the reversal of vertical positional nystagmus with head roll to either side in the monkeys and in the MdDS patients suggested that a primate analog of the human disorder had been created in the monkey by the roll while rotating. This included vestibular imbalance, as shown by the tendency of the patients to march to one side on the Fukuda stepping test, and the occasional spontaneous nystagmus, which was observed consistently in monkeys. In these subjects, the direction of the slow phases of the nystagmus and the direction of the postural deviation in the Fukuda test in the MdDS patients were always congruent, indicating that a vestibular imbalance had been created.

Long-lasting changes were never produced in monkeys by pitch while rotating, specifically implicating the roll system in the MdDS. Similar vertical nystagmus had also been previously produced in humans by extended exposure to a slow rotating room for several days in which the subjects intermittently made roll head movements. This induced vertical positional nystagmus for several hours thereafter when they rolled their heads (23, 24). Since only roll while rotating caused the abnormal eye movements in monkeys, not pitch while rotating, and similar vertical nystagmus with head roll was produced in humans (23), we postulated that the roll encountered during voyages was responsible for the generation of the MdDS (1).

It can be questioned whether there is a significant amount of roll especially on cruise ships that are the most common source of the MdDS patients. The stability of boats depends not only on the size of the vessel but also on the extent of the wind and waves as well as the direction of the boat’s progress. The sea is not always calm, and there are multiple reports of vessels being capsized in rough waters as reported by the Marine Accident Investigation Branch. A search of the literature revealed measurements of roll in the Bass Strait (between Australia and Tasmania) in a ship of 11,000 t deadweight, 603.7 ft in length and 77.6 ft in width (25). The ship had an average roll of ±6.3°, an average pitch of ±1.9°, and an average heave of 7.2 ft. The Bass Straight is 190 miles wide and 120 miles long. This was likely to have been very rough conditions, and the sea in cruises between Florida and the West Indies might generally be calmer. Additionally, there are roll stabilizers, i.e., planar strips of metal attached to the keel that can reduce roll. However, many although not all of the trips are in the Atlantic, and there can be strong weather that produces larger waves in any sea. Even large ships will roll if they encounter waves obliquely. Thus, there can be substantial roll, depending on the size of the waves despite the cruise ship’s roll stabilizers. While it is true that people walk around the deck in cruise ships, nevertheless, they are in stable positions for 6–8 h when they sleep at night or when they are sitting down for meals or in chairs to relax. Therefore, there can be adequate exposure to roll in cruise ships on the sea, particularly in heavy weather.

There have been no recent studies specifically on roll during flight in turbulent air, to our knowledge, although Dutch roll was described in light planes when banking in rough weather (26). Flutter of the wing tips and fuselage at 3–3.5 Hz has been generated in transport aircraft in turbulent conditions. See more details in the link provided (https://www.youtube.com/watch?v=kOBbAFzXrRg). Similar oscillations could contribute to the 0.3 Hz body oscillations in some MdDS patients after extended flights in turbulent weather.

**Figure 1:**
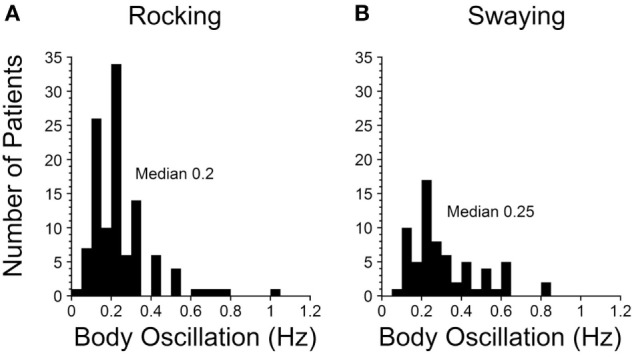
Frequencies of rocking **(A)** and swaying **(B)** in Mal de Debarquement Syndrome patients (2). The frequencies were determined on a Nintendo Wii board. The rocking frequencies were tightly centered around a maximum at 0.2 Hz, more for the rocking than the swaying. When there was no actual rocking or swaying, the perceived frequencies were determined with the elbow stabilized on a board, and the patient moved the forearm at the frequency of the perceived movement.

A striking finding was that there was a sharp peak in the average frequency of rocking and the perceived rocking in both our 2014 and 2017 papers at 0.2 Hz (Figure 1A). There was more spread in swaying (Figure 1B), probably due to the variation in determining the period of swaying, i.e., pitching was easier to observe and sense than swaying. The significance of the relatively conserved 0.2 Hz frequency (1 cycle/5 s) is that it signifies that a similar process is likely to be producing the rocking in virtually all of the MdDS patients after sea voyages. This implies that the frequency is being internally generated and is sensitive to an external stimulus of 0.2 Hz. The 0.2 Hz frequency is too slow to be produced by lesions of the inferior olive, since frequencies of such phenomena like palatal myoclonus typically have frequencies of about 1 Hz (27). Therefore, there should be another, separate source for the 0.2 and 0.3 Hz signals in the brainstem and cerebellum to account for the changes that are presumed to arise in the velocity storage integrator.

Modulation of roll depends heavily on the integrity of the cerebellar nodulus (28–31). This suggests that the nodulus may play an important part in the generation of the MdDS. In normal monkeys and humans, the orientation of the axis of eye velocity is always aligned with the spatial vertical or the gravitoinertial acceleration (GIA) during rotation, regardless of the position of the head in space (20, 21, 30–35). The underlying postulate for the generation of the MdDS was that the pitch orientation vector of the system had been transformed from its original position along gravity to a tilted position in roll (1). The purpose of the treatment was to bring the orientation vector back to the spatial vertical by activating the velocity storage integrator with an optokinetic stimulus that rotated around the spatial vertical. Thus, the proposed treatment was to roll the head of the affected subjects at the frequency of their perceived or actual rocking, swaying, or bobbing, while activating the velocity storage integrator with a low-frequency, constant velocity, full-field, optokinetic stimulus rotating around the spatial vertical against the direction of their vestibular imbalance. Presumably, this reoriented the integrator back to gravity or the GIA. If the correct direction of the vestibular imbalance was not chosen during the treatment, the body oscillations became worse, supporting the postulate that it was the conflict between the tilted orientation vector and the spatial vertical that had produced the MdDS.

As a result, it was possible to relieve the constant rocking, swaying, and/or bobbing in 70% of the original 24 patients after the second week of treatment, an improvement that was generally maintained in the original group when they returned home, and at follow-up after a year (1). In an additional 141 MdDS patients, the 2-week success rate was 80% (2); however, the rate of symptom reduction fell to 44% after 1 year, possibly due to stress experienced by these patients during long flights or rides home after treatment. This drop in efficacy must be addressed in future studies, but it should be emphasized that this was the first and only successful treatment for the MdDS.

The implication of these results is that the underlying cause for the MdDS is a disturbance in the control of roll at a specific direction and frequency, which is approximately 0.2 Hz after sea voyages and 0.3 Hz after turbulent flight. The fact that there was a difference in the sensed or actual oscillations implies that an external 0.2 or 0.3 Hz component of the roll on the sea or in the air was provoking the prolonged response to roll, but the highly specific oscillation frequencies across a wide range of MdDS patients strongly implies that those frequencies were recognized and perpetuated in the central nervous system. As will be shown below, we think that it is likely that the storage mechanism involves the nodulus of the vestibulocerebellum.

The treatment devised by Dai et al. (1) significantly reduced the symptoms by readapting the velocity storage mechanism to normality. This indicates that there had been no primary structural lesions in the vestibulocerebellar system that had caused the symptoms. This was essentially verified by the rapid return to normality, even after having suffered with the major symptoms for up to 20 years (2). However, the neural basis of the process that involved the velocity storage mechanism in the VOR has remained unclear. Here, a hypothesis composed of a number of postulates is proposed to explain how these findings are produced in the brainstem and cerebellum. First, however, we consider some of the requirements of such a proposal.

## Theoretical Demands of an MdDS Generation Hypothesis

The recurrent direction-changing nystagmus in periodic alternating nystagmus (PAN) after cerebellar lesions offers a potential scheme to explain the continuous sensations of rocking, swaying, and bobbing of the MdDS patients. PAN occurs after cerebellar lesions and causes a reversal of the direction of the slow and quick phases of nystagmus at frequencies of about once every 2–3 min (36). This has been interpreted as recurrent activation of groups of neurons on each side of the brainstem, i.e., as an adaptive process that can continuously reverse the direction of the nystagmus (37, 38). We have also reproduced the continuous reversal of the direction of horizontal nystagmus in the monkey by ablation of the nodulus and uvula (31, 39). In both studies, the recurrent cycle was terminated by an IM injection of the GABA_B_ agonist baclofen, similar to the effect of baclofen on the PAN (36). The PAN is, of course, considerably slower than the recurrent oscillations in the MdDS, but this analogy shows that there can be oscillation between neural groups on each side of the brainstem.

## MdDS Generation Hypothesis

Based on new findings from a three-dimensional study of the characteristics of vestibular-only (VO) neurons in the medial and superior vestibular nuclei (40), it is proposed that there is a similar situation that produces the MdDS, namely, oscillation between the VO neuronal groups on each side of the brainstem at frequencies of 0.2 or 0.3 Hz, controlled by output from the cerebellar nodulus that produces the MdDS.

## Relevant Questions

Previous studies of velocity storage have primarily been done on oculomotor aspects of vestibular activation, while the current interest is in head, neck, body, and leg movements or the perception of such movements. Therefore, there should be specific activation of the neural elements that would cause excitation of the body and limbs rather than the eyes. There should also be neurons that control different neural groups on either side of the brainstem to activate different parts of the body and limbs. If such an arrangement exists, these neural groups should have substantial connections between them that could monitor and maintain the oscillations. The VO neurons characteristically have a time constant during rotation of 15–25 s (40, 41), but these neurons are capable of responding at a much faster rate, i.e., up to 450 impulses/s (42, 43). As suggested earlier, there should be adaptable elements in these structures to account for the proposed shift in the pitch orientation vector that drives them into oscillation when confronted with exposure to head roll on the sea or in the air. Finally, there should be access to a 0.2 or 0.3 Hz signal from a structure that directly connects to these neural groups and sequentially drives them.

The VO neurons in the medial and superior vestibular nuclei meet these criteria. They are the neural structures that convert the time constant of the hair cells in the cupula of 4–4.5 s (42) into the VOR time constants of 15–25 s or longer (19–22, 44). The VO neurons receive direct input from the semicircular canals and output to the neck, body, and limbs through vestibulo- and reticulo-collic and vestibulo- and reticulospinal pathways (45–48). They have little direct output to the oculomotor system, and presumably contact the oculomotor system primarily through the VPS neurons (48), although there are also some direct vestibulo-oculomotor projections. As shown by Boyle and McCrea et al., the VO neurons reflect the imposed accelerations on the head and body, but do not go into action during volitional turns of the head (45–47). There are other vestibular neurons related to eye velocity that are activated during volitional head or head and body oscillations (49–51). Thus, there is a clear separation between the vestibular neurons that respond to passive head or head and body oscillations, as against a complex set of neurons in the vestibular nuclei that respond to visual input, volitional turns of the head or efference copy (45).

The VO neurons, however, are out of volitional control, and we postulate that the rocking, swaying and bobbing during the MdDS is generated by the VO neurons that similarly are not subject to efference copy, volitional control, or response of cortical or visual input. This is consistent with the finding during the MdDS that the patients have little or no active control of imposed rocking, swaying, and/or bobbing, or of the sensations associated with these movements. Of interest, drowsiness does not affect the rocking, swaying, or bobbing of the MdDS patients, and these oscillations or the perception of these oscillations persist even when the MdDS patients are not alert (Yakushin, Cohen, persoal communication).

The mixture of neurons that respond to passive head movement, voluntary head movement, and efference copy demonstrate that activity generated in the vestibular system can be overt or silent when viewed in terms of muscular activity. Thus, we assume that perceived rocking, swaying, and bobbing that is not observable in many of the MdDS patients does not mean that vestibulospinal and reticulospinal tracts are not active, but simply that the activity is not always manifest.

The VO neurons have extensive axonal connections to VO neurons on the other side of the brainstem and use GABA_B_ as the primary inhibitory agonist (52–54). Injections of baclofen caused a dramatic reduction in activity of VO neurons (22, 40, 55), and when the crossing axons were severed, velocity storage permanently disappeared (54, 56). Since we propose velocity storage is intimately involved in production of the MdDS (1), the disappearance of velocity storage when the connecting VO neurons in the brainstem were inhibited or severed supports our hypothesis that the VO neurons are involved in the production of the MdDS.

When examined in three dimensions, a majority of VO neurons on each side of the brainstem are primarily activated by rotation to the contralateral side and fail to respond to ipsilateral rotation (40). They also receive vertical canal and otolith inputs. Since these neurons project to the head, neck, body, and limbs through different components of the reticulospinal and vestibulospinal pathways, they each can activate a different set of head, neck, body, and limb movements, which could result in the rocking, swaying, and bobbing as well as the “gravity pulling” of the MdDS. Exactly how this is done is still not known, however.

## Evidence that the Orientation Vector can be Modified by Exposure to Roll

The experiments in monkeys using roll while rotating produced modification of the pitch orientation vector for up to 18 h (18). In other experiments, Eron et al. (57, 58) also have demonstrated that it is possible to condition the polarization vector of VO neurons by putting monkeys on their sides (in roll) for 30–60 min. This shifts the otolith polarization vector toward gravity in the side-down or rolled position. The shift in the habituated orientation of the neuron persisted for periods of several hours. Thus, the orientation of the VO neurons can be altered for substantial periods by exposure to roll. Changes in their polarization vectors, while not as profound, were also found in canal-related neurons that were located in the direct pathway of the VOR (59). Such changes could also be involved in the “gravity pulling” by altering the vertical canal and otolith inputs to the VO neurons.

## A Potential Cerebellar Source of the 0.2 or 0.3 Hz Body Oscillations

A large body of experimental data indicates that the nodulus and part of the uvula exert powerful control of the VO neurons and the velocity storage integrator. The VO neurons receive substantial input from the nodulus (31, 60–63). Pathways from the lateral portions of the nodulus cause disappearance of velocity storage and electrical stimulation of the nodulus, presumably activating these pathways, also causes a loss of velocity storage (64). This region of the nodulus is likely to discharge activity in velocity storage during visual suppression (19, 65) as well as loss of stored activity in velocity storage during “tilt-dumps” (20, 33, 39). In contrast, pathways from the central portions of the nodulus provide activity responsible for orienting the axis of eye velocity to the spatial vertical (20, 66). These functions are lost after nodulus lesions (30, 31). Habituation of the dominant time constant of the VOR is also controlled by the nodulus and is lost after nodulus lesions (67, 68). Therefore, there is extensive neural control of the VO neurons and of the velocity storage integrator through this structure (30, 31). Lesions of the nodulus also result in alternating nystagmus every 5 min (31, 39). Thus, the nodulus has a role in maintaining temporal adaptation of processes in the vestibular nuclei, that presumably can identify the source of the drive on neuronal groups in the brainstem that produce the PAN. Of note, this alternating nystagmus is eliminated by the action of baclofen, similar to the elimination of activity in VO neurons by the IM injection of baclofen (40, 55).

Nodulus lesions also cause a loss of roll eye movements and torsional nystagmus (28, 29), confirming the close association of the nodulus to roll. Thus, the tilted state of the pitch orientation vector in roll during the MdDS would be a natural function of the neural structure of the nodulus, as would the ability of the roll component of the nodulus to be modified by extensive exposure to roll on water or in the air.

Although the origin of the 0.2 and 0.3 Hz signal driving the VO neurons has yet to be discovered in monkeys or humans, this signal is present in the nodulus of the rabbit. In a comprehensive series of experiments, Barmack and Shojaku et al. (69–74) showed that there was a massive input from the vestibular nerve to the nodulus (73). A striking aspect of this is that about 70% of vestibular fibers in Scarpa’s ganglion project directly to the nodulus through the inferior olives, bypassing the vestibular nuclei. This input arises predominantly in the anterior and posterior canals that sense active or passive roll movements of the head and/or of the head and body. This activity is also transmitted through the medial and inferior vestibular nuclei to the inferior olives, where the individual planes of one anterior and the contralateral posterior semicircular canals are represented in individual neurons. The combined anterior and posterior canal activation in roll is also separately represented in another inferior olive nucleus (69). The combined activity that represents roll head and/or head and body in space is then transmitted by climbing fiber and mossy fiber inputs to the Purkinje cells in the contralateral nodulus. Thus, there is a powerful input to the nodulus continuously detailing the passive and active head and/or head and body movements in roll. Otolith neurons also sense the roll position of the head and/or the head and body relative to gravity and the GIA, and transmit this activity to the inferior olives and thence to the nodulus. The nodulus also receives a mossy fiber input from the dorsal cap of Kooy in the inferior olive that originates in the subcortical visual system in the nucleus of the optic track that carries optokinetic-generated activity to the vestibular nuclei and the nodulus.

**Figure 2:**
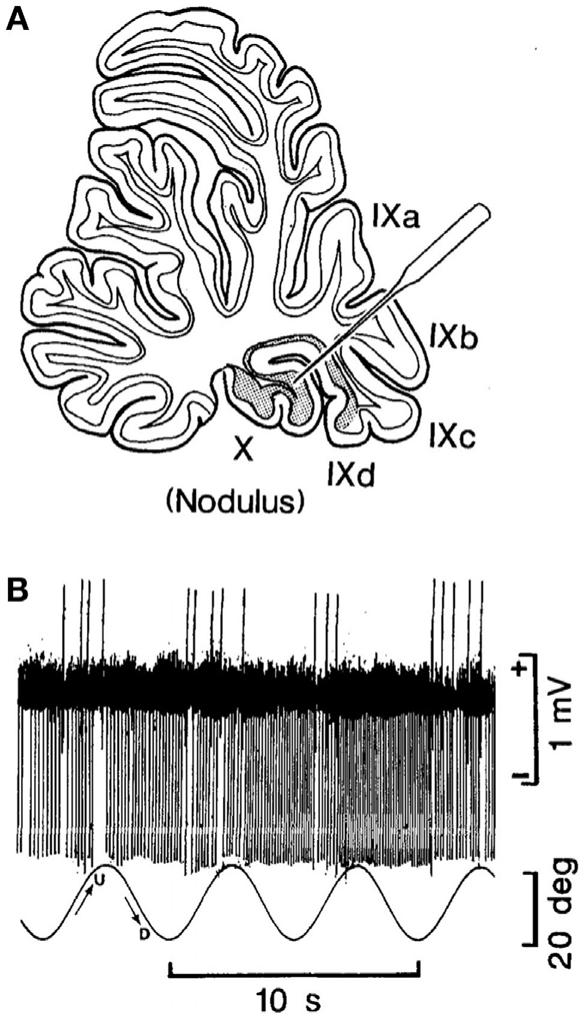
**(A)** Site of recording of the neuron of the nodulus shown in **(B)**. **(B)** Climbing Fiber-driven Purkinje cell activity from the site shown in **(B)**. The Purkinje cell fired three to five times. Each time, the animal was rolled into the left side-down position. The oscillation in roll is shown by the bottom trace. The oscillation amplitude is shown by the bar on the right, and the time base by the lowest trace. This figure is reprinted with permission. For further details, see the article by Barmack and Shojaku (70).

In their experiments, coordinated firing of the nodular and uvular Purkinje cells (Figure 2A) was produced by rolling the head and body statically and dynamically around the long axis at 0.2 Hz (Figure 2B). The 0.2 Hz oscillation in roll was chosen because it was the frequency that gave the best coordinated responses in the Purkinje cells on repeated testing at different frequencies of oscillation (Barmack, personal communication). The coordinated firing of the Purkinje cells at 0.2 Hz ceased in most cells, when the roll stimulus ended, and the cells returned to their irregular spontaneous activity. In about 15% of the Purkinje cells, however, the 0.2 Hz firing frequency faded and then returned for 300–400 s. Thus, it was possible to induce after-activity in some neurons at the preceding 0.2 Hz frequency that considerably outlasted the exciting 0.2 Hz roll oscillation (70). If such activity were present in the human nodulus, and if it were sufficiently prolonged, presumably it could supply activation of the output pathways to the VO neurons, and initiate the sense of swaying, rocking, and/or bobbing. Moreover, in a small number of Purkinje cells, it was possible to change the frequency of the after-activity to 0.3 Hz, as experienced in the MdDS after turbulent flight. A 0.1 Hz frequency was also induced that could be related to the spread in frequencies shown in Figure 1. They also encountered Purkinje cells that had a continuous 0.2 Hz firing rate that could maintain the preference of the Purkinje cells to oscillate at 0.2 Hz. Thus, there was activity in the rabbit nodulus and uvula that could have potentially caused activation of VO neurons that outlasted the roll stimulus that had induced the original activity. If this activity existed in humans, it could explain the frequency of the body rocking (Figure 1).

This work provides a potential neural basis for the 0.2 and 0.3 Hz oscillations in Purkinje cell firing that could be responsible for the 0.2 and 0.3 Hz oscillations in rocking, swaying, and bobbing of the MdDS patients. One may question whether results in rabbit as well as in monkey can be appropriately applied to humans. The rabbit’s eyes are centered ±85° from the midline of the head; whereas, the monkey and human have the fovea centered ±7° from the midline. Of course, there are many other differences between rabbits and humans. However, the vestibular system does not follow this rule. Baker and Straka and other colleagues have done extensive studies of the vestibular and oculomotor systems in fish and frogs (75–80). They note that “although the projections of the neurons vary among species, similar subgroups of major vestibular projection neurons originate from homologous segmental positions in the hindbrain of mammals, birds, and amphibians.”

Thus, it is striking that the semicircular canal to vestibular nuclei and inferior olive connections are very similar across mammalian species. For example, the angles of the planes of the semicircular canals and the insertions of the eye muscles driven by the semicircular canals lie in the same planes in humans, monkeys, goldfish, and sharks (81–83). Moreover, the eye movements induced by the canals are also the same across species in rabbits, dogs, cats, and monkeys (81, 82, 84). A striking example of the similar morphology of the end organs is that the planes of the semicircular canals are the same in monkeys and humans as in a brachiosaurus dinosaur that has been extinct for 155 million years (85). Of course, the dinosaur labyrinth is magnitudes bigger than the monkey labyrinth, but the planes of the canals are the same.

Baker and Straka conclude: “these comparative attributes among vertebrates suggest that, from basic wiring through function, the vestibular blueprint was established quite early during vertebrate evolution and, from the viewpoint of structure more than function, has been largely conserved throughout ~400 million years of vertebrate phylogeny (75).” From this, we postulate that the findings in the rabbit can be applied to monkeys and humans and that the driving frequency established in the nodular Purkinje cells are manifest in the VO neurons to produce the rocking, swaying, and bobbing of the MdDS in humans.

## Discussion

In this paper, we posit that the MdDS is produced by a deviation of the pitch orientation vector from the spatial vertical to one side in roll. Further, the deviation of the pitch orientation vector occurs as a function of contextual learning after prolonged exposure to roll on the sea or in the air. If the pitch orientation vector is displaced in roll, it should cause positional nystagmus. Consistent with this, many of the MdDS patients had a unique type of vertical positional nystagmus when their heads were put in roll on either side; the quick phases were up when the head was on one side and down when the head was on the other side (1). Most patients also had a vestibular imbalance, manifested by marching to one side in the Fukuda stepping test. The unusual vertical positional nystagmus was also produced in monkeys after extensive exposure to roll while rotating. This occurred in association with a vestibular imbalance manifested by spontaneous nystagmus in darkness (18). This provided the basis for recognizing that a similar process had produced the MdDS in monkeys as in humans. Such vertical positional nystagmus was also produced in humans after long exposure to a slow rotating room when they rolled their heads to the side (23, 24). The experiments in monkeys also demonstrated that such responses to roll while rotating only occurred in monkeys with a long VOR time constant, that considerably outlasted the 4.5–5.0 s input from the hair cells in the semicircular canals to steps of rotational velocity (42). Such time constants are produced in the velocity storage integrator, providing evidence that the MdDS was generated in velocity storage.

From this, it was postulated that the pitch orientation vector had been transformed from its original position along gravity to a lateral position in roll. It was further postulated that this shift had been produced by cross-coupling that had altered the position of the pitch orientation vector. The failure of pitch while rotating to cause a shift in the pitch orientation vector was interpreted as having strengthened, not modified, the pitch orientation vector. Model predictions were consistent with this hypothesis.

The finding that modification of the pitch orientation vector, i.e., its return to the spatial vertical, was produced by viewing low velocity, full-field optokinetic stimulation oriented around the spatial vertical confirmed this hypothesis. Thus, there was internal consistency between the conditions in both the MdDS patients and the response to roll while rotating. The ability to reverse the MdDS symptoms with a low velocity, full-field, optokinetic stimulus rotating against the direction of the vestibular imbalance, further strengthened the hypothesis that the optokinetic nystagmus (OKN) stimulus had countered the lateral tilt of the pitch orientation vector in roll. This conclusion was also supported by the disappearance of the rocking, swaying, and bobbing and the subjective symptoms after such treatment (1, 2).

## Evaluation of Specific Aspects of the Hypothesis

The mechanics of the basis for oscillation were also considered: namely, it was proposed that the MdDS is produced by repetitive oscillation of groups of VO neurons on either side of the medial and superior vestibular nuclei. These neurons project excitatory activity to muscles in the head, neck, body, and limbs that could produce the repetitive rocking, swaying, and/or bobbing or the sensation that these motions have occurred (40). This could explain why spontaneous nystagmus was not prominent in the MdDS patients. Instead they generally manifested their vestibular imbalance through the body and limbs, as evidenced by the lateral movement on the Fukuda stepping test. This would be expected if the primary site of activation of the MdDS movements was in the VO neurons that primarily project to the head neck, limbs, and body, and not to the oculomotor system. Rather, it is believed that the major projection of the VO neurons that are sensing body rotation is to the body, and not to the eyes (48). The proposed link to the oculomotor system from the VO neurons is through the VPS neurons, and these neurons become inactivated during drowsiness along with the eye movements, whereas the VO neurons continue their activity unchanged (13, 43). Similarly, in agreement with this, the perceived rocking, swaying, and bobbing continue even when the MdDS patients are drowsy. These findings are consistent with the clinical state and support the conclusion that the VO neurons are at the basis of the MdDS.

Finally, we propose that the signal driving the 0.2 and 0.3 Hz oscillations impinges on the VO neurons through projections from the nodulus. Since the nodulus has been shown to have a close association with roll (28, 29, 69–74), the exposure to repetitive roll while on the sea or in the air is presumably the trigger for the syndrome. As yet, the source of the 0.2 Hz signal, postulated to come from the nodulus, has not been found in humans or subhuman primates, but the findings by Barmack and Shojaku indicate that a preferred 0.2 Hz oscillation is present in the nodulus of the rabbit, and that the climbing fiber-driven Purkinje cells are readily excited by a 0.2 or a 0.3 Hz oscillation in roll. Such a signal may also be present in the human nodulus.

The same organization is probably also responsible for the generation of the commonly experienced Mal de Debarquement (MdD). It is likely that the less intrusive MdD is also produced by a transient shift of the orientation vector in roll, but fortunately, this is short-lived in most individuals. The underlying basis for the difference in durations of the MdD and the MdDS are not known, but presumably involve different tendencies for continued activation of nodulus Purkinje cells in the two conditions. Nor is it obvious why women are much more susceptible to development of MdDS than men. A similar propensity is also prevalent in migraine and motion sickness. If our postulate that the changes in the underlying frequency of nodular Purkinje cells is at the heart of the syndrome, then experiments in male and female monkeys and rabbits could prove interesting. It would also be important to determine if the effects of tilt of the body axis and exposure to brief flashes of light during recording of optokinetic after-nystagmus cause discharge in velocity storage (19, 65) Both of these functions have been demonstrated to originate in the nodulus (19, 30, 65).

## Possible Experimental Investigation of Unproven Assumptions

The postulate that the VO neurons were set into oscillation through an inhibitory link across the brainstem is presented without specific evidence that this actually occurs in the MdDS patients. For that reason, it would be important to have registration of VO neurons in monkeys with long VOR time constants after they had been exposed to several hours of roll while rotating that produced the vertical positional nystagmus when their heads were put in roll on either side of the midline. This preparation could also be useful in determining if the 0.2 Hz oscillation in the Purkinje cells in the rabbit were similar to those in primates. It is also possible to determine if the pitch orientation axis is aligned with the spatial vertical in recordings of neurons in the nodulus. If so, then it could be determined if the pitch orientation vector was tilted after generation of an MdDS analog in monkeys and whether it was possible to reorient the pitch orientation vector by exposure to a slowly moving, full-field OKN stimulus moving about the spatial vertical. It also might be possible to force a shift in the pitch orientation vector by exposure to roll while rotating, and to determine how much tilt of the OKN axis was sufficient to produce a tilt in the pitch orientation vector. Finally, it might also be possible to determine if the pitch orientation vector is actually strengthened after exposure to pitch while rotating (18).

Similarly, the hypothesis that exposure to an optokinetic stimulus oriented to gravity induces reversion of the pitch orientation vector could explain the finding that the body oscillations disappear briefly when the MdDS patients ride in cars (3–5). Presumably, some aspect of the visual streaming or the oscillations of the automobile temporarily restore the orientation vector back to the spatial vertical. This could be studied experimentally by blocking vision during the car rides, or by determining if rides on smooth, flat surfaces in well sprung cars fail to affect the sensations of the MdDS.

It would also be of interest to tilt the axis of rotation of the OKN when treating MdDS patients to determine whether tilts of the axis of the OKN stimulus failed to induce abolition of the MdDS, and if so, by how much tilt of the axis of rotation. The importance of the vestibular imbalance could also be studied by combining rotation at various velocities with the MdDS to determine whether it made the perception or actual oscillations better or worse, as does rotating the OKN stimulus during treatment against or in the direction of the vestibular imbalance. Finally, it would be of interest to reduce the time constant of the velocity storage integrator using the paradigm that was used to reduce velocity storage in motion sickness (66), and test the hypothesis that the MdDS could be improved by habituating or shortening the time constants of the VO neurons.

A critical experiment would also be to determine if the yaw axis orientation vector was tilted from the spatial vertical during off-vertical axis rotation (OVAR) in patients with the MdDS, and whether such a tilt reverted to its orientation to gravity after the patients had been successfully treated (20, 66). Such an experiment could provide proof of the tilted orientation vector hypothesis.

The syndrome considered in this manuscript is dependent on the presence of a velocity storage integrator and does not exist in monkeys and presumably in humans with a short VOR time constant. Velocity storage, as noted above, is not a recently developed phenomenon, since it is also present in the goldfish, evolved hundreds of millions of years ago (75). Ernst and Thomas have shown that it is possible to activate cross axis firing of neurons on each side of the vestibular nuclei of the goldfish with continuous rotation, as in humans (37, 38). The goldfish also have prominent cerebella with many similar connections as in mammals (75). Consequently, it could also be of interest if it were possible to produce cross-brain stem activation of vestibular units by roll while rotating in the goldfish. The point is that this type of cerebellar-driven oscillation of neurons in the vestibular nuclei may be a very old phenomenon. If so, then it would be of interest to determine if cerebellar-driven vestibular activity is an intrinsic phenomenon crossing species from fish to man.

## Qualifications to the MdDS Hypothesis

Two major qualifications could invalidate the hypothesis presented in this paper. First, there has been heavy emphasis on the speculation that the syndrome produced in the monkey by roll while rotating is essentially the same as that of the MdDS in humans. This was based primarily on the finding of abnormal positional nystagmus and a vestibular imbalance in both humans and monkeys. However, there were significant differences between these two that were encountered. Namely, there was more activation of spontaneous nystagmus in the monkeys than in humans in whom spontaneous nystagmus was rarely present. This suggested that the vestibulo-ocular component was larger in the monkeys than in humans. It could have been related to the differences in generation of the mdDS. The monkeys were rotated in yaw for several hours, whereas the humans presumably got their MdDS after prolonged exposure to roll, without the concomitant yaw axis rotation. More important, perhaps, was the difference in body movements. Rocking, swaying, and or bobbing was never observed in the monkeys after roll while rotating, but such movements or the perception of such movements were a cardinal feature of the MdDS. Of course, there was no manifest movement in many of the humans, only the sensation of movement, and it could not be ascertained whether the monkeys also had a sensation of movement, not manifest by rocking, swaying, and/or bobbing. If the VO neurons were driven by the nodulus, such activity would be expected. However, the monkeys studied in the 2009 paper were always chaired when out of their cages, so that it is possible that weak oscillations of limbs were never observed (18). If monkeys were to be used in Future studies, it would be important to have implanted EMG electrodes to determine if weak oscillations were present in the muscles after exposure to roll.

Second, heavy emphasis was placed on the origin of the role of the nodulus in perpetuating the body oscillations or the perception of the body oscillations. This postulate depended on data from the rabbit. However, there was little direct evidence that such activation of nodular Purkinje cells was also present in the individuals with the MdDS. If such activity does not exist in humans, then an important part of this hypothesis would be invalidated.

Despite these differences, the hypothesis that the pitch orientation vector had been tilted in roll that led to the treatment generated a therapy that was successful in a majority of the MdDS patients, for the first time (1, 2). If the study using OVAR can be performed, then it could potentially provide support for this portion of the hypothesis. However, these qualifications must be kept in mind in evaluating whether the hypothesis is generally valid.

## Other Treatment Necessities

A major criticism of the therapeutic results is that they were obtained without adequate controls. This largely accrued because there was no significant support for such a study. The patients were desperate for relief after having had the MdDS for years without relief, and some were even suicidal. Also, this was the first successful treatment for the MdDS, and the results in the initial study were statistically significant (1). Moreover, the patients were coming for treatment from all over the country and the world and were not able to return for motor studies without support. The fact that many of the patients had had their illness for many years without relief despite a wide range of investigative steps, extensive drug treatment, and prolonged physiotherapy without significant improvement rendered treatment, even without controls to be a necessity. Presumably, given the strong positive results, even though they were largely reported by telephone, provide the preliminary data to support a complete, controlled study. Such a study is now under consideration.

A significant problem remains in the treatment of the MdDS, namely, that there was a substantial reversion back to the rocking, swaying, and/or bobbing after treatment. This was generally attributed to oscillations during the ride home (2). Initial efforts to reduce this reversion with oral baclofen have not been successful. This might be due to its limited ability to cross the blood–brain barrier (86–89).

Intramuscular injections of baclofen in monkeys caused the disappearance of any vestige of velocity storage in the VO neurons (22, 40, 55). If our hypotheses that the MdDS is produced by VO neuronal activity are correct, suppression of VO neuron activity could stop the uncontrollable oscillations of the body during the MdDS. If the 0.2 or 0.3 Hz signals are coming from the cerebellum, however, we do not have the appropriate drugs to affect cerebellar circuitry, aside from the GABA_A_ and GABA_B_ inhibitory agonists, and more research is necessary on this subject.

Since cruises on the sea continue to be an attractive vacation, it is likely that we will continue to have numerous people who are afflicted by this malady. However, while it was originally considered to be untreatable, and people have even been driven to suicide by this condition, there appears to be the possibility of finally correcting the position of the pitch orientation vector so that it stays permanently on its appropriate orientation to the spatial vertical. Of course, adequate therapy demands reduction in the host of associated symptoms such as brain fog, sensitivity to sound and fluorescent lights, headaches, inability to work, depression, and suicidal tendencies that accompany the MdDS (2, 4, 6, 7, 9, 10, 13–15), but this must be addressed in detail when the uncontrolled body movements or the sensation of these movements ceases.

## Author Contributions

All authors contributed to the generation of this manuscript.

## Conflict of Interest Statement

The authors declare that the research was conducted in the absence of any commercial or financial relationships that could be construed as a potential conflict of interest.

## Abbreviations

G, gravity; GIA, gravitoinertial acceleration; GABA, gamma amino butyric acid; Hz, per second; MdD, Mal de Debarquement, also sometimes known as “Land sickness”; MdDS, Mal de Debarquement syndrome; OKN, optokinetic nystagmus; PAN, periodic alternating nystagmus; VO neurons, vestibular-only neurons; VPS neurons, velocity-pause-saccade neurons; VOR, vestibulo-ocular reflex.
